# Round the Hospitals

**Published:** 1918-07-06

**Authors:** 


### ROUND THE HOSPITALS.

Every circumstance of treachery and long-drawn-out barbarity attended the destruction of the *Llandovery Castle*, news of which came as a crushing blow to the Canadian rejoicings on the national fête day. The ship was chartered by the Canadian Government to convey their sick and wounded from England to Halifax. She was on the return journey and had no wounded on board, but fourteen nursing sisters and eighty members of the Canadian Army Medical Corps, all of whom, except Major Lyon, Sergeant Knight, and four orderlies, are believed to have perished. The German submarine had every right under the Hague Convention to stop and search the ship, and could have done this with-

**ROUND THE HOSPITALS—(*continued*).**

out incurring risk. Her commander, however, decided to sink it without inquiry. Not content with this, he manœuvred to ram the boat in which were the twenty-four survivors, and there is reason to fear that the twelve shells subsequently fired in the direction of the wreckage were responsible for sinking many of the other boats. Only ten minutes elapsed between the firing of the torpedo and the sinking of the *Llandovery Castle*. The boat in which were the fourteen nursing sisters was seen to be sucked under when the vessel sank, and there is no hope that any of them were saved. As evidence of deliberate determination to kill unarmed men and women whom Germany has solemnly pledged herself to hold immune from injury this outrage stands out as the blackest yet on record against a dishonoured nation.

As we go to press the bells of St. Paul's Cathedral will be sounding a note of triumph over the severance from the Mother Country of the thirteen American colonies under the Declaration of Independence July 4, 1776. Doctors and nurses have taken their full share in the reversal of public opinion which has turned this once melancholy anniversary of mistaken and defeated policy into a symbol of harmony between free nations. Long before the multitudinous energies of the States began to exert an influence in other directions in this country, her hospital and nursing methods were known and admired here. We are proud to reflect that our columns have now for many years past afforded readers an opportunity of gauging the medical, nursing, and philanthropic successes of America. We have much to learn from her, perhaps still something to teach. In celebrating with her the Day of Independence we look forward to new triumphs over disease and pain to be accomplished through union of aims, against the common enemy—spiritual wickedness in high places.

Dominion Day, July 1, the fifty-first anniversary of the creation of the Dominion of Canada, was kept this year with particular spirit in the many Canadian hospital centres in this country. At Cliveden Mrs. Waldorf Astor entertained a large company of distinguished visitors, among whom were Miss Macdonald, the Matron-in-Chief of the Canadian Military Nursing Service, and Matron Caines. A splendid programme of sports rejoiced the 600 patients, nearly all of whom were able to be present, and the nursing staff, which numbers 81, with forty of Queen Mary's Army Auxiliary Corps, gave indefatigable help. It was a typical Canadian celebration in an old English setting, graced by a real summer's day. For the moment war was forgotten. But as the afternoon wore to its close a sinister rumour spread through the gay crowds, only too soon to be confirmed by official tidings.

The thirty-first annual meeting of the Royal National Pension Fund for Nurses, held on June 27, 1918, Sir Everard Hambro, K.C.V.O., in the chair, brought out some memorable facts. The nurses who hold policies in this Fund, the greatest provident fund for women workers in the world, are now holders of £1,034,000 of British Government Stocks, which yield a revenue of 5 per cent., and are to be paid off at par. Two thousand three hundred and eighty-two nurses are now in receipt of annuities at the rate of £65,000 a year. They have reason to bless themselves for the provision they made for their old age. During the sixth quinquennium of the Fund, ended December 31, 1917, the invested funds have increased by a total amount of £365,639, being an average rate of increase of £73,000 for each year of the quinquennium. The invested funds now exceed two millions sterling, and on the whole investments of the Royal National Pension Fund for Nurses, after deducting income-tax, the average rate of interest for the past year was £4 3s. 6d. per cent., being 8s. 3d. above the rate for 1916; that is an increase of more than £8,000 in the Pension Fund's interest receipts for 1917, compared with those for 1916. Continuously since the beginning of the present year evidence has been afforded of the tendency to increase the salaries and fees paid to British nurses. This tendency, which has need of considerable growth, should enable the nurses to put more and more money into the Pension Fund, for their own protection and security. Sir Thomas Dewey, whose great experience in insurance and saving associations constitutes a record, expressed the great pleasure he had had in proclaiming far and wide the fact that, no professional class in the United Kingdom has done so much to provide for their own future as the nurses, for they have saved and invested upwards of two millions of their money to provide for annuities for their later years. We may expect, as we certainly hope, that the importance of saving and thrift will more and more impress itself upon all working nurses, and that they will use the Pension Fund as a liberal banker, safeguard, and friend for them in every phase of life which it may be their fate to encounter and overcome. Once a woman begins to save she continues the practice with commendable tenacity, with results for herself that have often been really magnificent. This truth the Fund has demonstrated frequently during the upwards of thirty years of its useful existence.

Mr. Hayes Fisher opened the present week, which, as everyone knows, is dedicated to His Majesty King Baby, by trying to cheer his hearers with the news that the birth-rate, though low in this country, is lower still in Germany, and that while the infant mortality for urban districts in England and Wales is 104 per 1,000, it is 138 for urban areas in German. We decline to be comforted by any such comparison. The figures are a disgrace to us, and it does not help in the least to know that we are sinning in very bad company. It is better news that the President of the Local Government Board was able to promise that before this month is out the Maternity and Child Welfare Bill is to become law. Local authorities are awaiting its provision of a 50 per cent. subsidy from

**ROUND THE HOSPITALS**—(*continued*).

the Treasury for the inauguration of many important services essential to the preservation of infant life.

THE Colonial Nursing Association, at a meeting held at the Imperial Institute on the 3rd inst., Viscount Gladstone in the chair, unanimously and wisely decided to change its name to that of the Overseas Nursing Association. This change is calculated in our judgment to add to the popularity of the Association, to increase the demand by nurses to become members of its staff, and generally to further the best interests of our Overseas Dominions. The report acknowledges the indebtedness of the Association to the Colonial Office for the assistance which has been given in obtaining passages and passports for private nurses. The total number of nurses serving abroad during the year has been 298, of whom 65 have been working as private nurses, 13 in hospitals not under Government, 14 under the Government of Western Australia, 8 under the Government of the Union of South Africa, and 198 in Government service in the Crown Colonies. C.N.A. nurses home on leave have been temporarily employed in Military hospitals. 19 additional silver badges for meritorious service of five years and upwards have been awarded. Two nurses, Miss M. Graham, from Southern Nigeria, and Miss M. Poulter, who were passengers on the ss. *Abasso* and ss. *Appapa*, respectively, when these ships were torpedoed, lost their lives. By recommendation of the Sub-Committee, in co-operation with the Duchess of Devonshire, and, as far as possible, with the Royal Victorian Order, arrangements are in progress for assisting in the supply of nurse midwives in the outlying districts of Canada in compliance with the wishes of the Canadian authorities. This work will be under the direction of a newly-appointed Dominions Committee constituted under Rule 12, and made responsible to the Executive Committee. Persons having special knowledge of the Dominions are being invited to join the Dominions Committee, and its meetings will be held at the offices of the Overseas Nursing Association. Freedom is reserved to act with responsible Canadian or other public bodies desiring a more progressive policy, and in other Dominions similarly. An assistant secretary has been appointed especially for the Dominions Committee's work, all correspondence going first to the secretary. We congratulate the Overseas Nursing Association on its new policy and development. It is greatly indebted to Mrs. Carnegie and Lady Piggott for the devoted honorary service they have rendered, and to the tactful policy pursued by the president, the Right. Hon. Viscount Gladstone, G.C.B.

SIR ARTHUR STANLEY introduced the purposes of the College to his hearers in a trenchant speech. The Mayor had reminded the meeting that Derby was Florence Nightingale's own county, and as treasurer of St. Thomas's Hospital the speaker took occasion to look back to the creation of the Nightingale Training-school as the nation's memorial to the founder of modern nursing after the Crimean War, and to look forward to the endowment of the College of Nursing, after the present war, for the consolidation of the nursing profession. They wanted at least £100,000 for this purpose; they wanted new premises, having long outgrown their present ones, but were not going to sink money in building. Miss Sparshott, who was formerly matron of the Derbyshire Royal Infirmary, spoke with energy and conviction of the big things at which the College was aiming. She believed that membership would broaden the nurse's outlook, make for unity of purpose, and lead to the study of the body corporate rather than of the individual. All augurs well for the development of a thoroughly live Centre in Derby.

THE Guildhall, Derby, was the scene of a memorable meeting on June 27, when the Hon. Sir Arthur Stanley, G.B.E., M.P., and Miss Sparshott came down to address a crowded audience of nurses and others on the aims of the College of Nursing. The chairman was the Mayor of Derby, and there were present Lord Roe, of Derby; Mr. Arthur Cox, president of the Derbyshire Royal Infirmary; Mr. F. W. Greaves, chairman of the Derbyshire Children's Hospital; Dr. Laurie, Dr. Barwise, Dr. Macphail, and others. Miss E. M. Sutcliffe, matron of the Derbyshire Royal Infirmary, was the convener of the gathering, and must have been gratified by the cordial response to the invitations issued, and by the enthusiastic resolve to form a Centre of the College at Derby, which was the practical outcome of the meeting.

SHEFFIELD has also had good meetings in support of the local Centre of the College of Nursing. A thoroughly representative gathering of nurses assembled at the Cutlers' Hall, Sheffield, on June 28 to hear an address by Miss Cowlin, organising secretary of the College, with the result that many new members of the Centre were enrolled. The chair was taken by Mrs. Stephenson, who spoke of the need among district nurses for some such centre of union as the College afforded. Miss Cowlin urged on all present the value of organisation for professional purposes and the need for establishing a sound legal and educational basis for the nursing profession. A second meeting was held at the War Hospital, Wharncliffe, in the evening, at the invitation of the matron, Miss Scudamore Smith, R.R.C., when many members of the Queen Alexandra Imperial Military Nursing Service were able to be present.

ALL hospital workers and friends in the nursing world will be glad to hear that Miss L. V. Haughton, late matron of Guy's Hospital, has found Surrey air in the midst of the pines and heather very beneficial, and looks forward to being at work again next year, for she has made strides towards health and strength during 1918 especially. His Majesty the King has hoped continuously that Miss Haughton might have been

**ROUND THE HOSPITALS**—(*continued*).

able to attend a public Investiture, but her continued ill-health has prevented this. It was, therefore, a gratifying surprise, and a gracious action on the part of His Majesty, that Dame Ethel Becher, G.B.E., should have gone down especially to the pines and the heather on the 19th ult., for the purpose of presenting to Miss Haughton the Royal Red Cross (First Class) which was awarded to her in March 1917. We may all look forward with pleasurable anticipation to Miss Haughton's restored health and return to work during 1919. May God's blessing attend her throughout life!

THE Welsh Hospital, Netley, has seen an amazing development in a comparatively short time under its able matron, Miss Evans, now retiring, and has become one of the biggest war hospitals in the kingdom. It owes much to Miss Evans's tireless energy and gifts of organisation, and the affection and gratitude of her staff and of the medical officers connected with it have been manifested by a handsome presentation on her departure. Lieut.-Colonel Cook, R.A.M.C., in presenting her with the gold wrist-watch and album subscribed for among them all, declared that she had never failed in her duty or neglected the interests of her staff. It is indeed a great test to have watched over so speedy an expansion of the hospital, and to have so conducted its nursing administration during these anxious times that the standard of nursing has been well maintained throughout. Miss Evans will be succeeded by Miss Kathleen Stewart, details of whose career we give on p. 314.

WE have often had occasion to deplore the jaundiced views on English nursing matters which have been set before the readers of the *American Journal of Nursing*. It is therefore with considerable satisfaction that we note in the June issue of this otherwise well-edited paper the prominence given to an able and dignified letter from Miss Lloyd Still and Miss Hughes on the misapprehensions about the College of Nursing to which the readers have been exposed for want of accurate information. The large number of nurses from the States who now pass through London and meet nurses from our great training-schools have their own opportunities of judging the accuracy of Miss Lavinia Dock's impression of English nursing politics. But it was due to the close alliance between nurses on both sides of the Atlantic to set before the nursing world in America the ideals which animate the promoters of the College and the causes why it has met with so wholehearted a welcome from English nurses. A rhapsody contributed by Miss Dock on those who have so persistently misled her is the foreign editor's only and not very impressive reply to the arguments which expose her ignorance of the facts.

VERY interesting details of the nursing staff are afforded in the recently issued report of Edinburgh Royal Infirmary for the year 1916-17. Out of a nursing staff numbering 293 (exclusive of four temporary probationers), sixty certificated nurses and twenty-seven probationers left during the year, while two nurses and eighty-six probationers joined the staff. Of those who left, forty-one joined the military service, and only three took up private nursing. It has been ascertained that at least 350 nurses holding the Royal Infirmary certificate have been employed in military hospitals, 150 having gone direct from the wards. Miss Millar, matron of the 2nd Scottish General Hospital; Sister Laing, acting assistant matron No. 20 General Hospital, France; and Sister Murray, No. 26 General Hospital, France, have received the Royal Red Cross; Sister Gardner, Egypt, and Miss Heriot, Assistant Lady Superintendent of Nurses, have been made Associates of the R.R.C. Applications for training continue numerous, 650 as against 843 the previous year. The daily percentage off duty for illness was 3.11. A badge for the trained nurses of the school was instituted last year. The design is taken from the hospital arms and shows the pelican feeding its young; it is presented to nurses on gaining their certificates, and 200 former nurses have applied to purchase it.

SOME interesting posts as laboratory assistants are offered in hospitals in France, which the V.A.D. headquarters at Devonshire House are endeavouring to fill. Training in hospital is not essential, but there must have been some experience in laboratory work. The rate of pay is 39s. 6d. per week, from which 14s. is deducted for board and lodging. Candidates must be over twenty and of good physique. If selected for service, they will be sent over to France at once.

BECKENHAM COTTAGE HOSPITAL had Pound Days on June 13 and 14, and realised over £35 in money and nearly 500 lb. of groceries, besides 28 lb. of soap and various other gifts. The school children took a leading part, and their big collection of gifts was escorted to the hospital in a wheelbarrow. One child gave his margarine ration for the week, another two eggs laid by his own hen, and many contributed vegetables from their own allotments. Altogether a fine rally.

FOOD Orders this month include the following:—July 13.—*Sugar.*—No supplies thenceforward to be permitted to Welfare Centres at which children merely attend for medical or other advice. July 14.—No article of food to be served or supplied in any hut, hostel, canteen, or buffet which has not been licensed by the Food Controller. *Rationing.*—National scheme comes into operation; new ration-books in force. *Tea.*—New distribution scheme in force. July 16.—*Early Potatoes.*—Reduction to 2d. per lb. Vegetarians are now entitled to one extra fats coupon per week on surrender of their meat-card. Jews who for religious reasons do not eat bacon may obtain coupons in exchange entitling them to extra fat rations on signing a special declaration form.

**For A New Charter of Liberty, "Ierne" and Nurses All, see p. 308;**
**The Training of an Asylum Matron, p. 307; Matrons' and Sisters' Appointments, p. 314.**

